# Preparation of Graphite-UiO-66(Zr)/Ti electrode for efficient electrochemical oxidation of tetracycline in water

**DOI:** 10.1371/journal.pone.0271075

**Published:** 2022-08-09

**Authors:** Bicun Jiang, Fuqiang Liu, Yang Pan, Yan Tan, Chendong Shuang, Aimin Li

**Affiliations:** 1 State Key Laboratory of Pollution Control and Resources Reuse, School of the Environment, Nanjing University, Nanjing, PR China; 2 Nanjing Innovation Center for Environmental Protection Industry Co., Ltd., Nanjing, PR China; University of Wollongong, AUSTRALIA

## Abstract

Tetracycline (TC) is widely-used antibiotic pollutant with high toxicity, refractory, persistence and bacteriostasis, and its removal from water needs to be enhanced. In this work, a novel Graphite-UiO-66(Zr)/Ti electrode was successfully prepared and evaluated for electrochemical oxidation degradation of TC. The electrochemical performance tests indicate the Graphite-UiO-66(Zr)/Ti electrode had higher electrochemical oxidation activity, which achieved higher TC removal efficiency (98.1% ± 1.5%) than Ti plate (65.2% ± 3.5%), Graphite-MIL-53(Al)/Ti electrode (79.5% ± 2.9%) and Graphite-MIL-100(Fe)/Ti electrode (89.0% ± 2.6%). The influence of operating condition was also systematically studied, and the optimized condition was pH 5.0, 20 mA/cm^2^ current density and 0.1 M electrolyte (Na_2_SO_4_). Through the liquid chromatography mass spectrometry (LC-MS), the TC degradation pathway by Graphite-UiO-66(Zr)/Ti electrode oxidation was proposed. Under the •OH free radical oxidative decomposition effect, the double bond, phenolic group and amine group of TC were attacked. TC was transformed into intermediate product ① (m/z = 447), then was further degraded to intermediates ② (m/z = 401) and ③ (m/z = 417). The latter was fragmented into small fractions ④ (m/z = 194), ⑤but-2-enedioic acid (m/z = 116) and ⑥oxalic acid (m/z = 90, the proposed intermediate). In addition, TC removal remained at 89.6% ± 2.7% in the sixth cycle of operation, which confirmed the efficient reusability and stability for antibiotics removal from water.

## Introduction

Tetracycline (TC) has been widely used in therapeutic medicine and animal husbandry [1, 2]. The annual production of TC has reached 97,000 tons in China, accounting for almost 50% of total antibiotic production [3, 4]. Due to the human improper treatment and animals’ poor digestion, TC has been widely present in various environmental medias including wastewater, groundwater, surface water, soil and sediment [5–7]. The TC concentration is 0.1–4.5 μg/L in surface water and groundwater [8, 9], while it’s up to 96–1300 ng/L in wastewater [10]. Moreover, the TC concentration in water shows a growing trend. TC has the characteristics of high toxicity, refractory degradation, persistence and bacteriostasis [1, 2, 11, 12], causing serious threats to the environmental safety and human health. Therefore, it is necessary to remove TC in water environment.

Many methods are used to remove TC in water, including biological methods, coagulation, sedimentation and adsorption. However, these conventional methods have the disadvantages of cumbersome operation, low removal efficiency and secondary pollution [13–15]. Electrochemical oxidation has received more attention for its better effectiveness, simple operation, mild conditions and environmental friendliness [16, 17]. It is considered to be an efficient technology to remove TC [5, 16]. For the electrochemical method, the material of electrode is the key to the electrolysis performance. Traditional anodes such as carbon electrode have the shortcoming of poor performance and stability [18]. Hence, many advanced materials were developed as anode for electrochemical oxidation of pollutants. Boron-doped diamond (BDD) is the most representative type of anode, which has strong oxidation, outstanding pollutant removal performance and excellent stability [7, 19]. BDD anode had high degradation rate (> 95%) of TC within 4 h, but its high cost limited its industrial application [7]. Ti electrode was frequently employed as the electrode substrate for electrolysis due to the low cost, good conductivity and favorable electrochemical performance. The terbium doped Ti/IrO_2_, Ti/RuO_2_-IrO_2_, Ti/Ti_4_O_7_ and Ti/SnO_2_-Sb_2_O_3_/PbO_2_ anodes for electrochemical removal of TC all have achieved excellent TC removal rates higher than 95% within 3–6 h [19–23].

Metal organic frameworks (MOFs) are a kind of crystalline microporous material, which has multi-purpose catalytic activity, significant structural diversity, high specific surface area and adjustable pore size. MOFs are widely used to treat wastewater as photocatalytic and adsorption materials [24–26]. However, MOFs are considered to be the electrode materials with poor electronic conductivity and dispersibility. Therefore, high electron-conductive carbon material such as Graphite could be employed for the composite electrode [27, 28]. Samarghandi et al. [29] doped Graphite into PbO_2_ anode, and the removal rate of MB was increased by 27.9%. Therefore, Graphite-MOFs composite electrode was expected to have great potential application on the electrolysis to contaminants removal. However, this type of electrode was rarely reported for water treatment.

In this work, three different MOFs, MIL-53(Al), MIL-100(Fe) and UiO-66(Zr) were synthesized by hydrothermal method and then employed to the preparation of electrode. Graphite and the MOFs were combined to fix on Ti substrate face to form the Graphite-MIL-53(Al)/Ti, Graphite-MIL-100(Fe)/Ti and Graphite-UiO-66(Zr)/Ti electrodes. MOFs and Graphite-MOFs/Ti electrodes were characterized, respectively. The TC removal efficiencies by the different electrodes were evaluated under different pH, current densities and electrolyte (Na_2_SO_4_) concentration. In addition, the degradation mechanism was proposed by identification of free radicals and intermediates.

## Material and methods

### Materials

TC, N-dimethylformamide (DMF), terephthalic acid, sodium sulfate, tert-butanol (TBA), oxalic acid, methanol (MeOH), 1,4-benzoquinone (BQ), sodium hydroxide, acetone, zirconium chloride (ZrCl_4_), aluminium nitrate (Al(NO_3_)_3_) and Ferric chloride (FeCl_3_) were all analytical grade and purchased from Aladdin (Shanghai, China) without any purification treatments. Polystyrene (PS, extrusion grade, G1919229) and graphite powder (mesh = 250) were purchased from Aladdin (Shanghai, China). The Ti plate (purity > 99.9 wt%) was bought from Baoji Hongxinyuan (China). Prior to modification, the Ti plate was pretreated according to the following procedure [5]: Firstly, it was polished by sandpaper (600 mesh), then etched by oxalic acid solution with a mass fraction of 10% at 98°C for 2 h; afterwards, the etched electrode was put in a mixture of acetone: NaOH solution (1 mol/L, volume ratio = 1:1) for 1 h and then rinsed with deionized water.

### MOFs synthesis

UiO-66(Zr), MIL-100(Fe) and MIL-53(Al) were synthesized by the hydrothermal method according to the previous literature with slight modifications. Briefly, for the synthesis of UiO-66(Zr): ZrCl_4_ (0.54 mM) and terephthalic acid (0.75 mM) were ultrasonically dissolved in 15 mL of DMF, then the mixture solution was transferred into a Teflon-lined autoclave and heated at 120°C for 48 h [30]; for the synthesis of MIL-100(Fe): FeCl_3_ (0.01 M) and terephthalic acid (0.01 M) were ultrasonically dissolved in 50 mL of DMF. After that, the mixture solution was transferred into a Teflon-lined autoclave and heated at 150°C for 24 h [31]; for the synthesis of MIL-53(Al): Al(NO_3_)_3_ (1.0 mM) and terephthalic acid (0.5 mM) were ultrasonically dissolved in 50 mL of DMF. Next, the mixture was transferred into a Teflon-lined autoclave and heated at 220°C for 72 h [32]. When the autoclave cooled down to room temperature, the as-prepared MOFs powders were washed with DMF and ethanol consequently for three times in order to remove the residual solvents, then dried at 80°C overnight in a vacuum oven.

### Electrode preparation

Firstly, PS was dissolved in DMF and employed as the coating adhesive to fix MOFs on electrode [33]. Then a certain number of as-prepared MOFs was added into the PS solution. Meanwhile, graphite was also incorporated to increase the electrode conductivity [34]. The mixture was ultrasonically shaking for 20 min, following by continuous stirring for 1 h. Afterwards, 100 μL of the mixture was evenly coated on the surface of the titanium plate electrode (active surface area = 4 cm^2^), and then dried and solidified at 80°C. Finally, the other side of the titanium sheet electrode was also coated to fabricate the Graphite-MOFs/Ti electrodes according to the same operation mentioned above. The photo of Graphite-MOFs/Ti electrodes was provided in supporting information (S1 Fig).

### Characterization

The morphology of the as-prepared MOFs and Graphite-MOFs/Ti electrodes were examined by scanning electron microscopy (SEM, Hitachi, S-4300 SE, Japan). Fourier transform infrared (FT-IR) spectra were measured by FT-IR spectrometer (Nicolet NEXUS670, ThermoFisher, USA) with a resolution of 4 cm^-1^. The powder X-ray diffraction (XRD, D/max 2500/PC target X-ray diffractometer, Rigaku, JPN) was carried out using Cu Ka radiation (λ = 0.1541 nm) in a scanning range of 3–60° at rate of 2°/min. X-ray photoelectron spectroscopy (XPS, Escalab Xi^+^, Thermo Fisher Scientific, USA) was employed to analyze the chemical states of electrode. The pore size distribution and specific surface area of MOFs were measured by MicrotracBEL equipment (ASAP 2020 HD88, Micromeritics, USA). Thermogravimetric analysis of MOFs and Graphite-MOFs/Ti electrodes was conducted by thermogravimetric analyzer (TGA, Pyris 1, PE, USA). The temperature ranged from 30°C to 800°C with heating rate of 10°C/min.

### Electrochemical test

A standard three-electrode system was used to evaluate the electrochemical properties of the prepared electrodes with an active area of 4 cm^2^. Electrochemical measurements were carried out on an electrochemical workstation (CHI 660D, Shanghai Chenhua, China). The Ti electrodes without and with the decoration of MOFs were used as the working electrode, while a saturated calomel electrode (SCE) and a platinum electrode served as the reference and counter electrode, respectively.

Cyclic voltammetry (CV) analysis was performed at a scan rate of 100 mV/s in the 0.1 M Na_2_SO_4_ solution with the addition of TC (100 mg/L). Linear sweep voltammetry (LSV) analysis was conducted for the prepared electrodes to evaluate the oxygen evolution potential (OEP) in 0.05 M Na_2_SO_4_ solution at a scan rate of 1 mV/s. Chronoamperometric tests were used to study the electrocatalytic response of TC in 0.05 M Na_2_SO_4_ solution at a constant electrode potential (+1.8 V).

### Electrochemical degradation

Electrochemical degradation of TC (100 mg/L) solution containing a certain concentration of electrolyte (Na_2_SO_4_) was conducted in a cylindrical reactor with a direct current power supply (Dahua Instrument, Beijing, China). The fabricated Graphite-MOFs/Ti electrodes were used as anode with an effective area of 4 cm^2^ and the graphite was used as cathode. The gap between anode and cathode was 2 cm. The influence of pH (3–8), current density (5–30 mA/cm^2^) and electrolyte concentration (0.02–0.15 M) on the antibiotic’s removal (Eq 1) was investigated.

$$\mathrm{Antibiotic}’s\;\mathrm{removal}\;(\%)=(1‐\frac{C_{}}{C_{0}})\times 100\%$$ (1)

where *C*_*0*_ and *C* were concentrations (mg/L) of antibiotics without and with electrocatalytic oxidation for time *t* (min), respectively.

The removal kinetics of TC were fitted with pseudo-first-order model, which was expressed in Eq 2.


$$\ln(\frac{C_{}}{C_{0}})=‐\mathrm{kt}$$ (2)

The stability of fabricated Graphite-MOFs/Ti electrode was determined by determining the removal rate of tetracycline for 6 cycles. Moreover, the electrodes after electrocatalysis were characterized by FTIR and SEM as mentioned above.

### Free radicals’ identification

The effect of free radicals on the electrocatalytic oxidation of TC was investigated. MeOH (25 mM), TBA (25 mM) and BQ (5 mM) were used as the scavengers of SO_4_^-^•, •O_2_^—^and •OH free radicals [35]. The removal efficiency of antibiotics was studied without and with the addition of scavengers.

### Other analysis

The concentration of TC was measured by high performance liquid chromatography (HPLC, 1260 system, Agilent, USA) equipped with a UV detector at wavelength of 355 nm [36] and Purospher RP-18 column (5 μm, 25 cm × 4.6 mm). The methanol/acetic acid (V: V = 9: 1) mixed solution was used as the mobile phase with flow rate of 1.0 mL/min and the injection volume was 20 μL.

In order to determine the degradation pathway, a liquid chromatography mass spectrometry (LC-MS) (6550 Q-TOF, Agilent, USA) equipped with a extend C18 column (1.7 μm, 2.1 × 50.0 mm) was used to identify the intermediate products of TC after oxidative degradation. Formic acid/acetonitrile mixed solution was used as the mobile phase at a flow rate of 0.3 mL/min. The identification of intermediate was carried out in the ESI positive ion mode with the gas temperature of 150°C and the sheath gas temperature of 350°C. The flow rates of sheath gas and the drying gas were 12 and 15 L/min, respectively. The m/z range and ion spray voltage were 100–1000 and 4500 V, respectively.

## Results and discussions

### Characterizations

**Fig 1:**
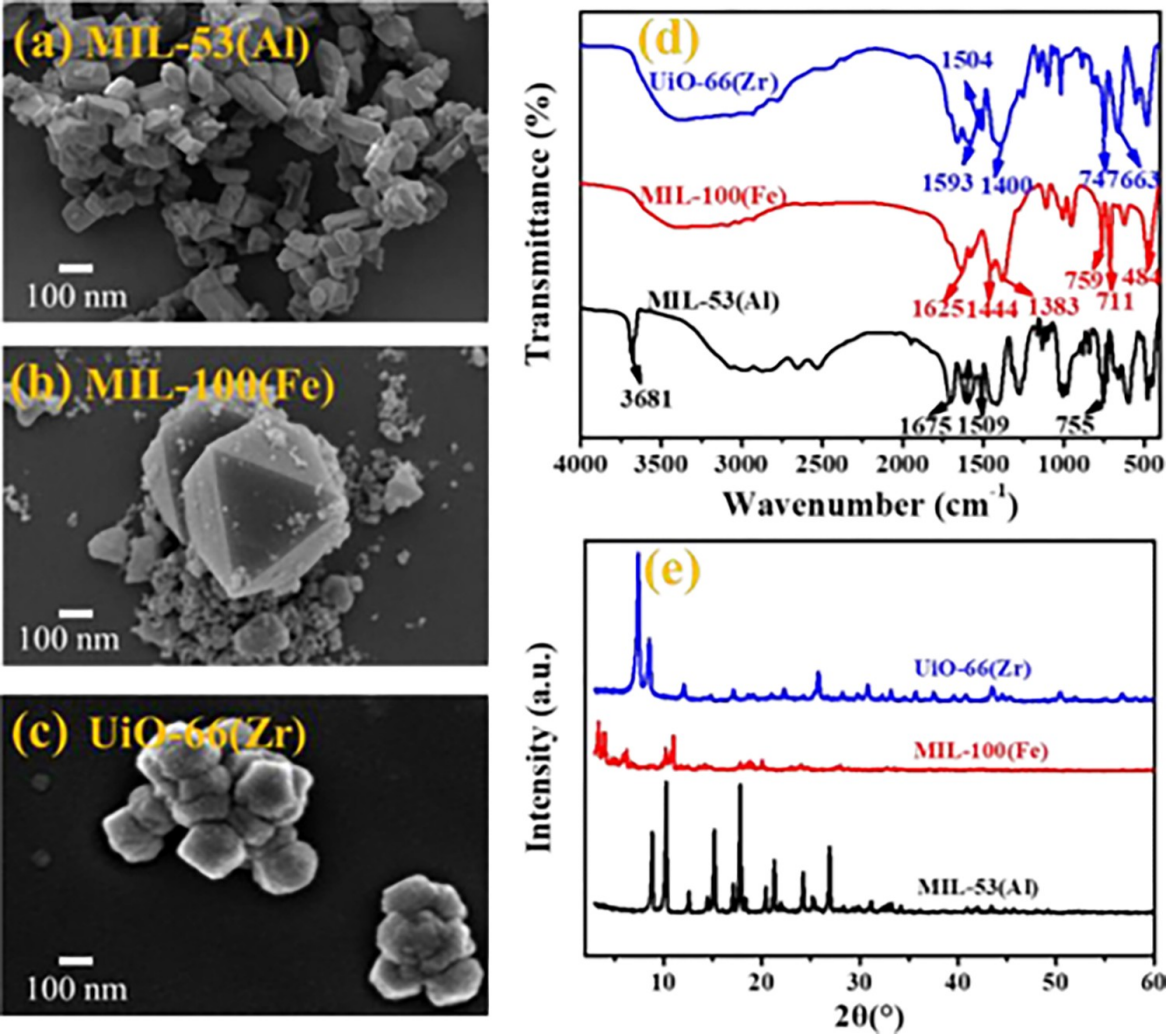
Characterization of MIL-53(Al), MIL-100(Fe) and UiO-66(Zr). (a-c) SEM images. (d) FTIR spectra. (e) XRD patterns.

The SEM images of MIL-53(Al), MIL-100(Fe) and UiO-66(Zr) were observed in Fig 1A–1C. The crystals of MIL-53 (AL), MIL-100 (Fe) and UiO-66(Zr) -MOF synthetic frameworks were irregular polyhedral particles, octahedron and uniform hexagon, respectively, which was consistent with the previous research results [37–39]. The FT-IR spectra of MOFs (Fig 1D) presented the typical absorbance reported in previous literature for these materials. UiO-66 showed two peaks at 1593 and 1400 cm^-1^ corresponding to the asymmetric and symmetric stretching of O-C-O in organic binder, respectively, while they shifted slightly for the other two MOFs [40]. Meanwhile, a strong peak that located at 1504 cm^-1^ corresponded to the vibration of C = C in benzene ring for UiO-66(Zr) and MIL-53(Al). The peaks of MIL-53(Al) and MIL-100(Fe) at 711, 755 and 759 cm^-1^ corresponded to the bending vibration of C-H in benzene. In addition, the peaks around 747 and 1444 cm^-1^ were attributed to the bending vibration of OH, and the peaks around 663 and 1509 cm^-1^ were owing to the bending vibration of O-C-O in organic binder [41]. XRD patterns (Fig 1E) further displayed the main characteristic diffraction peaks of MIL-53(Al), MIL-100(Fe) and UiO-66(Zr), which were consistent with the crystalline structure of MOFs reported by Du et al. [32, 42, 43]. These characterizations confirmed that the MIL-53(Al), MIL-100(Fe) and UiO-66(Zr) were successfully synthesized in this study.

**Fig 2:**
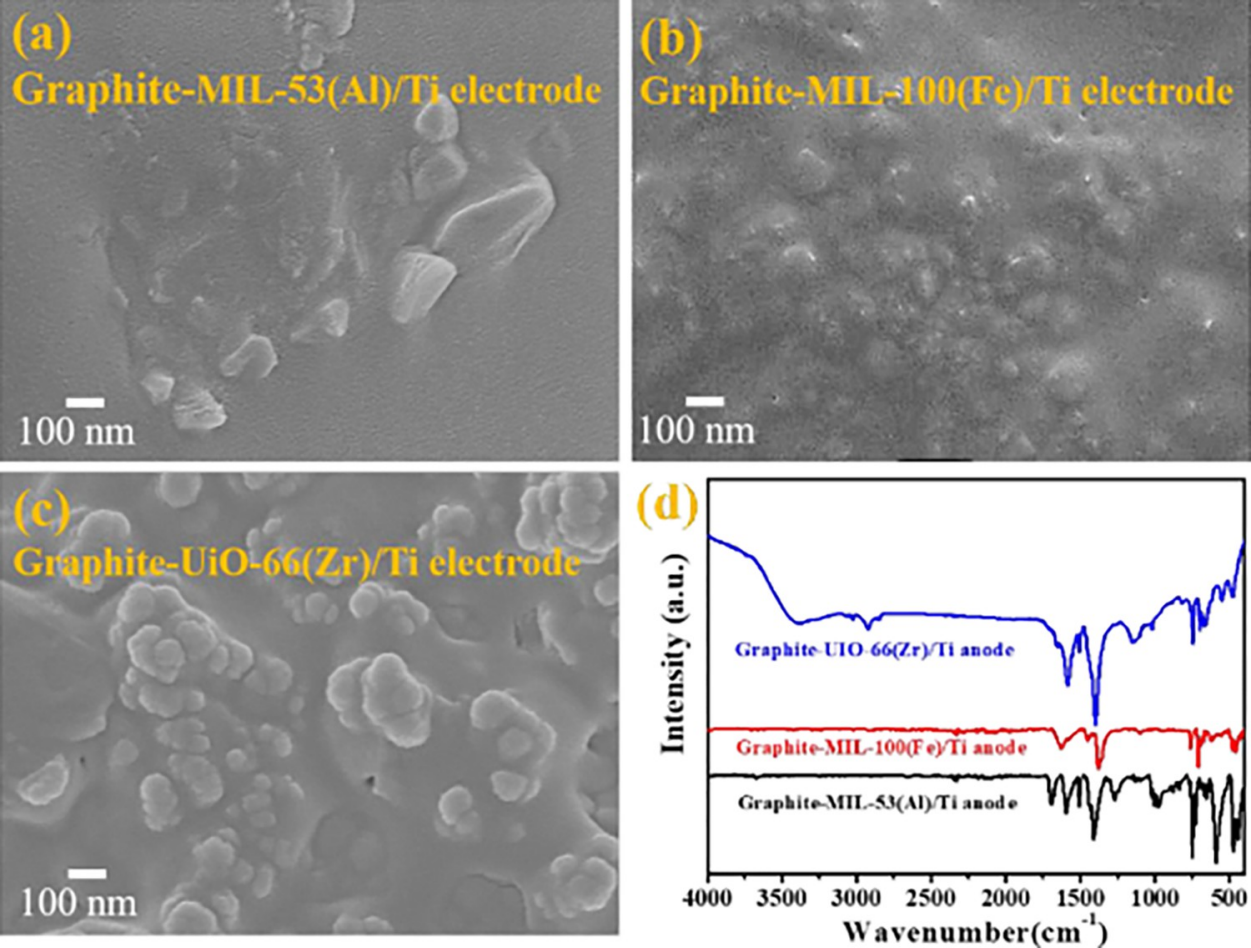
Characterization of electrodes. (a-c) SEM graphs of Graphite-MOFs/Ti electrodes. (d) FTIR spectra of Graphite-MOFs/Ti electrodes.

**Fig 3:**
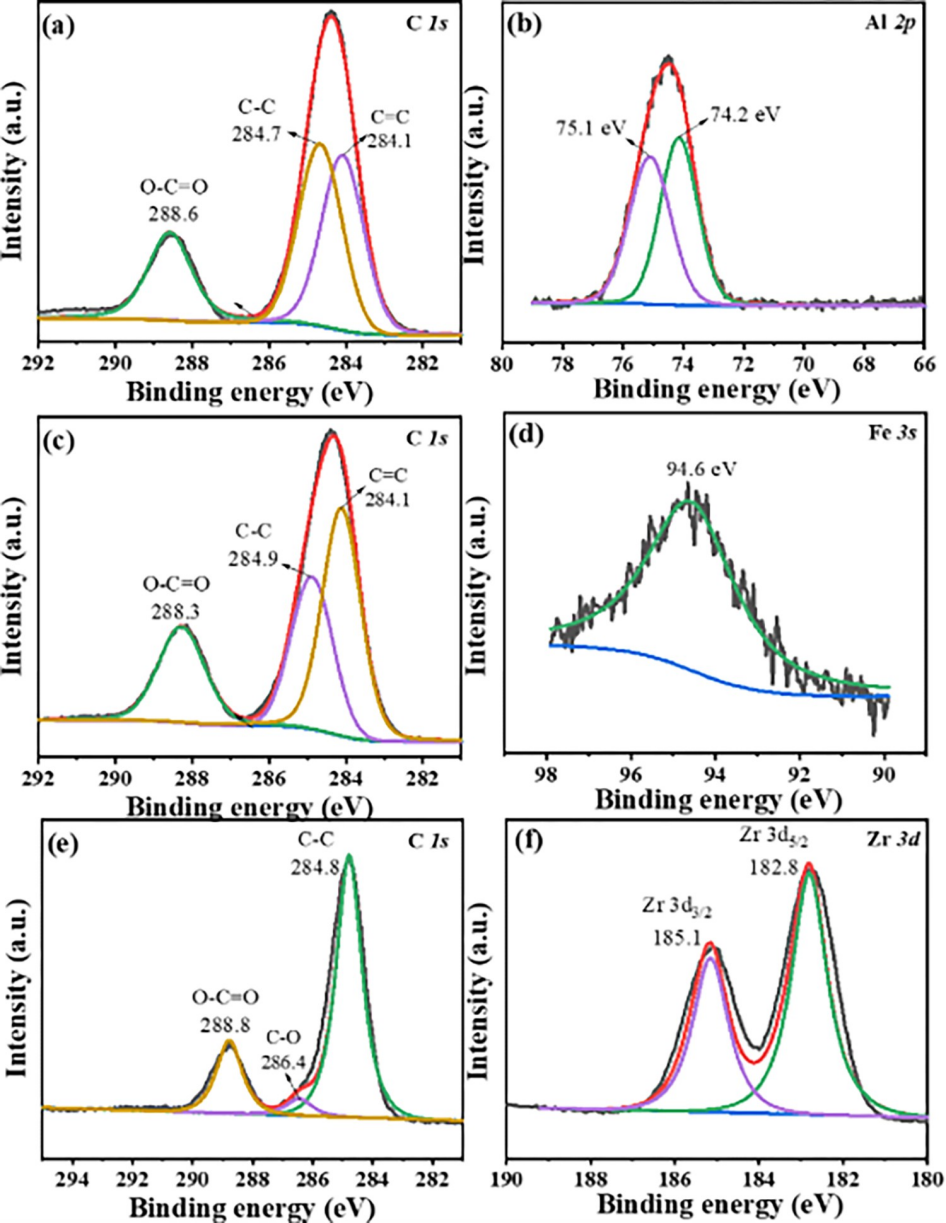
High-resolution XPS spectra. (a-b) Graphite-MIL-53(Al)/Ti electrode. (c-d) Graphite- MIL-100(Fe)/Ti electrode. (e-f) Graphite-UiO-66(Zr)/Ti electrode.

The prepared Graphite-MIL-53(Al)/Ti, Graphite-MIL-100(Fe)/Ti and Graphite- UiO-66(Zr)/Ti electrodes were characterized, as shown in Fig 2. According to the SEM graphs (Fig 2A–2C), MIL-53(Al), MIL-100(Fe) and UiO-66(Zr) nanoparticles were evenly distributed on the surface of the corresponding Ti plate, respectively. The FTIR spectra (Fig 2D) illustrated the characteristics peaks of organic binder in MOFs (1400–1600 cm^-1^) on Graphite-MOFs/Ti electrodes, suggesting the successful blending of MOFs on Ti electrode. The result was consistent with others work [31, 33, 44]. The XPS survey spectra showed the presence of C and O elements in the three electrodes (S2 Fig). Meanwhile, Al, Fe and Zr were found for Graphite-MIL-53(Al)/Ti, Graphite-MIL-100(Fe)/Ti, and Graphite-UiO-66(Zr)/Ti electrodes, respectively. Fig 3 depicted the high-resolution XPS spectra. The characteristic peaks of C = C, C-C and O-C = O originated from organic binder for C 1s spectrum were all observed at Graphite-MIL-53(Al)/Ti (284.1, 284.7 and 288.6 eV), Graphite- MIL-100(Fe)/Ti (284.1, 284.9 and 288.3 eV), and Graphite-UiO-66(Zr)/Ti (286.4, 284.8 and 288.8 eV) electrodes. Moreover, the Al 2p spectrum had two major peaks for Graphite-MIL-53(Al)/Ti electrode, 2p_1/2_ at 75.1 eV and 2p_3/2_ at 74.2 eV. The Fe 3s spectra of Graphite- MIL-100(Fe)/Ti revealed that the main peak was at 94.6 eV and the peaks of Zr 3d (Zr 3d_3/2_ 185.1 eV and Zr 3d_5/2_ 182.8 eV) emerged on Graphite-UiO-66(Zr)/Ti electrode. These results confirmed the successful preparation of the Graphite-MIL-53(Al)/Ti, Graphite-MIL-100(Fe)/Ti and Graphite-UiO-66(Zr)/Ti electrodes.

### Electrochemical properties of electrodes

**Fig 4:**
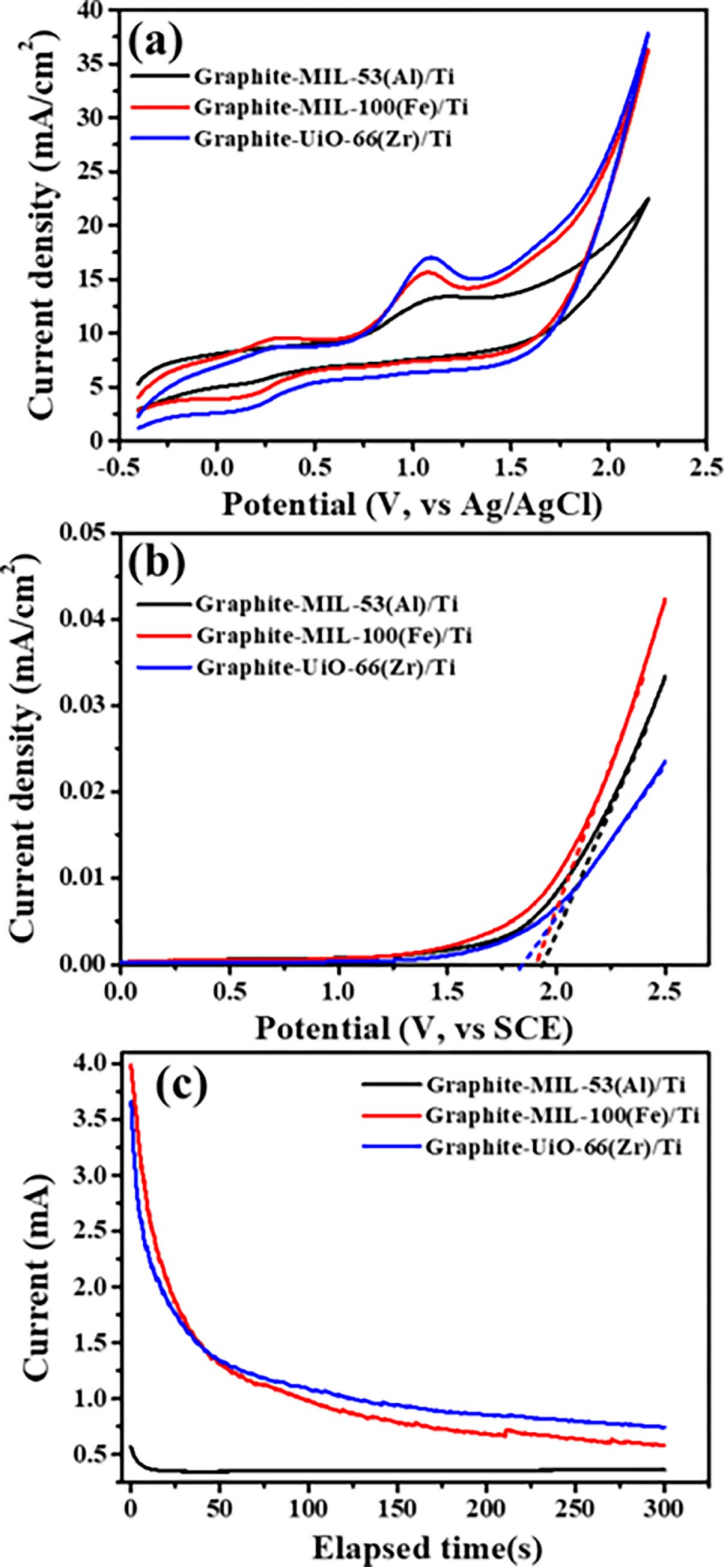
Electrochemical tests of the prepared electrodes. (a) CV response in 100 mg/L TC solution with 0.1 M Na_2_SO_4_, scan rate 100 mV/s. (b) LSV analysis in 100 mg/L TC solution with 0.05 M Na_2_SO_4_, scan rate 1 mV/s. (c) Chronoamperometric tests in 100 mg/L TC solution with 0.05 M Na_2_SO_4_.

The electrochemical property of the as-prepared electrodes was determined by CV, LSV and chronoamperometric tests. As verified in Fig 4A, the oxidation peaks for degradation of TC by the as-prepared electrodes were 1.08–1.13 V. Graphite-UiO-66(Zr)/Ti electrode had higher oxidation peak current density of 16.96 mA/cm^2^ than others (Graphite-MIL-100(Fe)/Ti electrode: 15.46 mA/cm^2^; Graphite-MIL-53(Al)/Ti electrode: 12.91 mA/cm^2^), suggesting that Graphite-UiO-66(Zr)/Ti electrode had higher electrochemical activity to oxidize TC. In addition, we also determined the oxygen evolution reaction (OER) potential of anodes by LSV analysis (Fig 4B) and they were 1.94, 1.90 and 1.83 V, respectively, for Graphite-MIL-53(Al)/Ti, Graphite-MIL-100(Fe)/Ti and Graphite-UiO-66(Zr)/Ti electrode. The lower OER potential means that more reactive oxygen species generated, which was conducive to the degradation of pollutants [35, 45]. Chronoamperometric tests (Fig 4C) also revealed the highest current of Graphite-UiO-66(Zr)/Ti electrode in steady state conditions over others, which could favor the degradation of pollutant. In general, the electrochemical performance of Graphite-UiO-66(Zr)/Ti electrode was slightly better than that of Graphite-MIL-53(Al)/Ti electrode, but significantly better than that of Graphite- MIL-100(Fe)/Ti electrode.

### TC degradation performance

**Fig 5:**
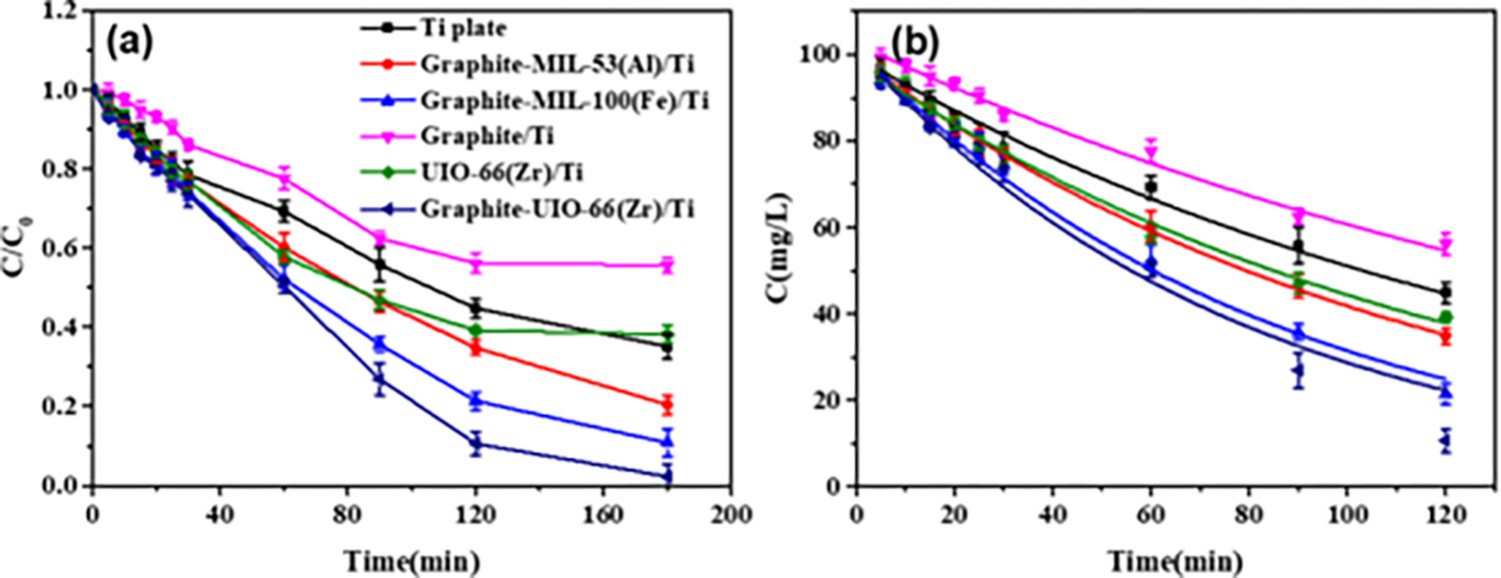
The degradation performance of TC by all electrodes under current density of 25 mA/cm^2^. (a) TC removal in 100 mg/L TC solution with 0.1 M Na_2_SO_4._ (b) the removal kinetics using pseudo-first-order model.

The prepared electrodes were applied to remove TC under a current density of 25 mA/cm^2^. As shown in Fig 5A, it could be seen that TC concentration sharply decreased at the first degradation stage until it reached equilibrium after 180 min. Graphite-UiO-66(Zr)/Ti electrode efficiently removed TC with the highest TC removal efficiency (98.1% ± 1.5%) due to its highest electrocatalytic activity, while the TC removals for the Ti plate, Graphite-MIL-53(Al)/Ti electrode and Graphite-MIL-100(Fe)/Ti electrode were 65.2% ± 3.5%, 79.5% ± 2.9% and 89.0% ± 2.6%, respectively. The removal efficiency was only 1.1% after degradation by Graphite-UiO-66(Zr)/Ti electrode without electricity (S3 Fig), indicating the TC degradation was mainly attributed to the electrolysis rather than the effect of adsorption. In addition, the removal efficiencies of TC degraded by Graphite/Ti electrode and UiO-66(Zr)/Ti electrode were 44.3% ± 2.5% and 61.7% ± 1.1% respectively (Fig 5A), which were much lower than that of Graphite-UiO-66(Zr)/Ti electrode. Therefore, the composite of Graphite and UiO-66(Zr) on Ti plate could promote the degradation of TC. The degradation kinetics of TC by the electrodes were also determined by fitting the plot of concentration with degradation time using pseudo first order model. As depicted in Fig 5B, TC removal fitted well with the kinetic model and the corresponding parameters were illustrated in Table 1. The degradation rate (*K*, min^-1^) of Graphite-UiO-66(Zr)/Ti electrode (0.01263 min^-1^) was significantly higher than others (0.00668, 0.00871, 0.01173, 0.00525 and 0.00798 min^-1^, respectively for Ti plate, Graphite-MIL-53(Al)/Ti, Graphite-MIL-100(Fe)/Ti, Graphite/Ti and UiO-66(Zr)/Ti).

**Table 1:** Parameters of the pseudo-first-order kinetic model for electrochemical degradation of TC. (100 mg/L, 0.1 M Na_2_SO_4_, 25 mA/cm^2^, 120 min).

Electrodes	*K* (min^-1^)	*R* ^2^
Titanium plate	0.00668	0.99251
Graphite-MIL-53(Al)/Ti electrode	0.00871	0.9992
Graphite- MIL-100(Fe)/Ti electrode	0.01173	0.9943
Graphite/Ti	0.00525	0.9900
UiO-66(Zr)/Ti	0.00798	0.9951
Graphite-UiO-66(Zr)/Ti electrode	0.01263	0.9723

### Factors influencing TC removal

**Fig 6:**
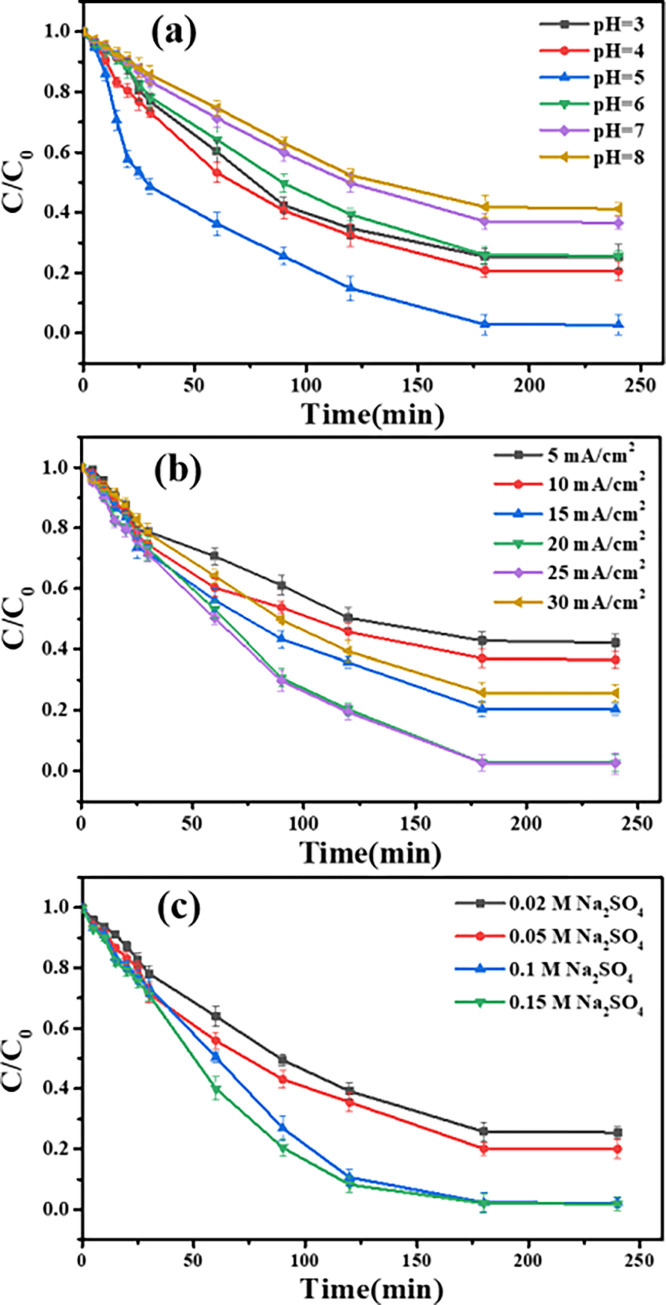
Influencing factors of TC removal by Graphite-UiO-66(Zr)/Ti electrode under current density of 20 mA/cm^2^. (a) Effect of pH. (b) current density. (c) electrolyte (Na_2_SO_4_) concentration. (100 mg/L, 240 min).

The effect of operating conditions of pH, current density and electrolyte (Na_2_SO_4_) concentration on TC removal was also explored. Firstly, as shown in Fig 6A, the TC removal achieved the largest level higher than 97% with the pH of 5.0, whereas the further decrease in pH lowered the TC removal to 74.7% ± 4.1%. The decrease may be attributed to the reason that TC has positive charge and electrically repulsed with the positive electrode, which was detrimental for pollutants to be adsorbed on the active sites on electrode [46] and then deteriorated TC removal. As the pH increased to 8, it would boost the consumption of electrolyte and lowered the conductivity of solution [46], thereby decreasing the TC removal (58.8% ± 2.2%). The result was consistent with the reported results from Tang et al. [47]. Secondly, Fig 6B showed the variation of TC removal under different current density. As expected, with the applied current density increased from 5 to 20 mA/cm^2^, the TC removal increased from 57.8% ± 2.8% to 97.3% ± 2.7% after 180 min. The result was attributed to the improvement of charge transfer with the increased current density, thereby enhanced the organic molecules decomposition [48]. Nevertheless, if the current density was higher than 25 mA/cm^2^, the TC removal decreased to 74.1% ± 2.9% due to the promoted side reaction of hydroxyl radicals [49]. Additionally, Fig 6C illustrated the variation in TC removal under different electrolyte (Na_2_SO_4_) concentration. The TC removal increased from 74.5% ± 2.1% to 97.8% ± 1.5% with the increasing electrolyte (Na_2_SO_4_) concentration, while this increase was not obvious when the electrolyte concentration was higher than 0.1 M. There could be an explanation for the decrease that lower or higher electrolyte concentration could cause the poor conductivity and reduce the generation of hydroxyl radicals, thereby decreasing the TC removal [48]. Consequently, considering TC removal efficiency and energy consumption, the conditions for TC removal of pH 5.0, 20 mA/cm^2^ and 0.10 M Na_2_SO_4_ by Graphite-UiO-66(Zr)/Ti electrode were optimized.

### Degradation mechanism

**Fig 7:**
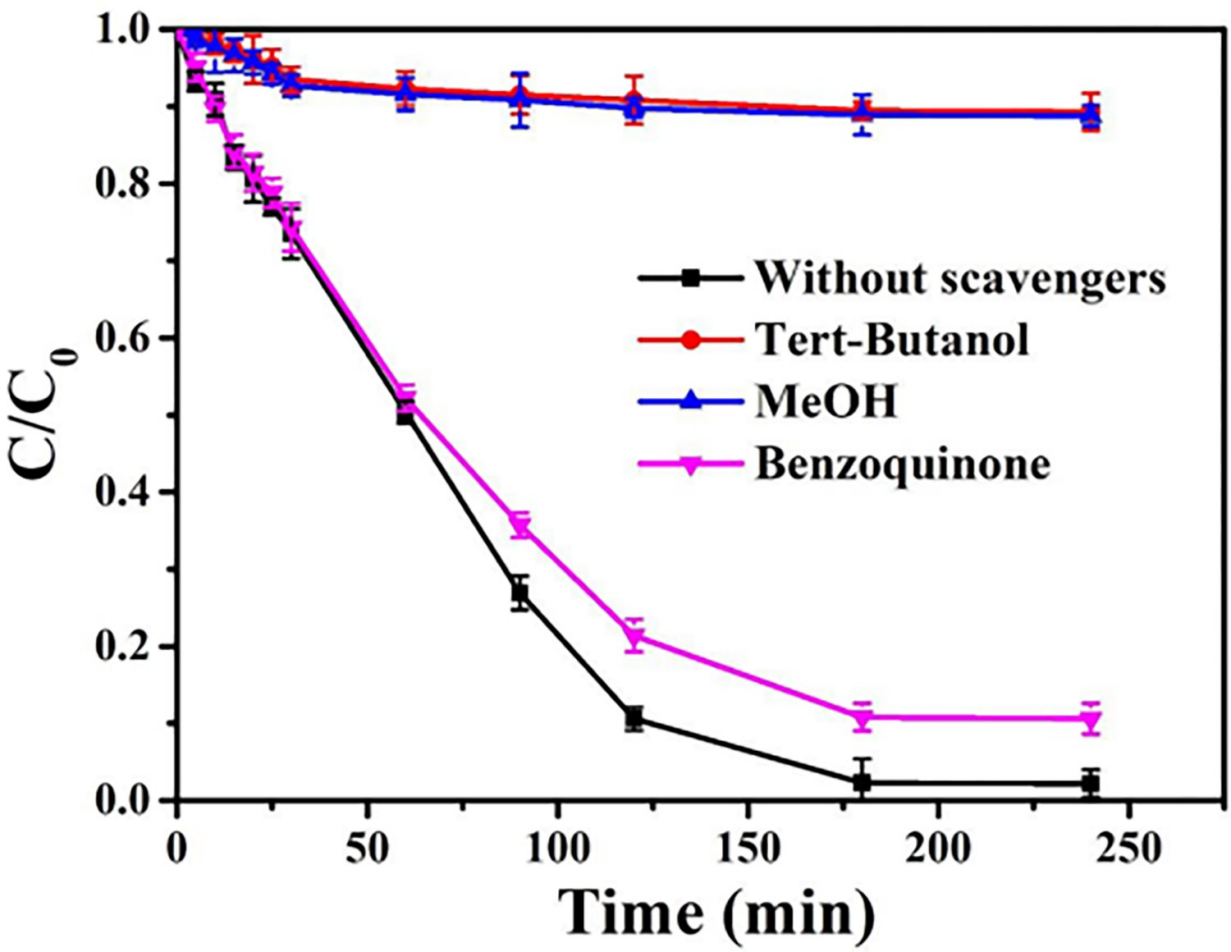
Effect of scavengers of free radicals on TC removal under current density of 20 mA/cm^2^. (100 mg/L, 0.1 M Na_2_SO_4_, 240 min, pH = 5.0).

In the electrolysis in aqueous media, free radicals generated on electrode and oxidized TC during electrochemical oxidation process [50, 51]. Hence, effect of free radicals on TC removal was determined by the addition of scavengers in order to clarify the removal mechanism. TBA and MeOH were considered as radical scavengers of •OH and SO_4_^–^•. MeOH had a high reaction rate with •OH and SO_4_^–^•, while TBA reacted faster with •OH than SO_4_^–^• [52]. After adding MeOH, the removal rate of TC was not significantly different from the removal rate when adding TBA (Fig 7). This confirmed that •OH free radical played a key role in TC removal by the electrode, while there was no relationship between SO_4_^–^• and TC removal. In addition, BQ was the radical scavenger of •O_2_^—^and •OH [53, 54]. The elimination of •OH by TBA and MeOH reduced the removal of TC by 85.5%, while BQ only reduced by 7.7%. The result indicated that the reaction was mainly caused by •OH, rather than •O_2_^—^. This mechanism was different from the bisphenol A removal by Co_3_O_4_-Bi_2_O_3_ catalysts, in which SO_4_^–^• played a dominant role [55]. Moreover, free radicals that played a major role in the degradation of TC were different under different pH. Huang et al. also found a similar mechanism at pH 6.0, while •O_2_^—^free radical played a key role in TC removal when the pH value was 3.0 [56].

**Fig 8:**
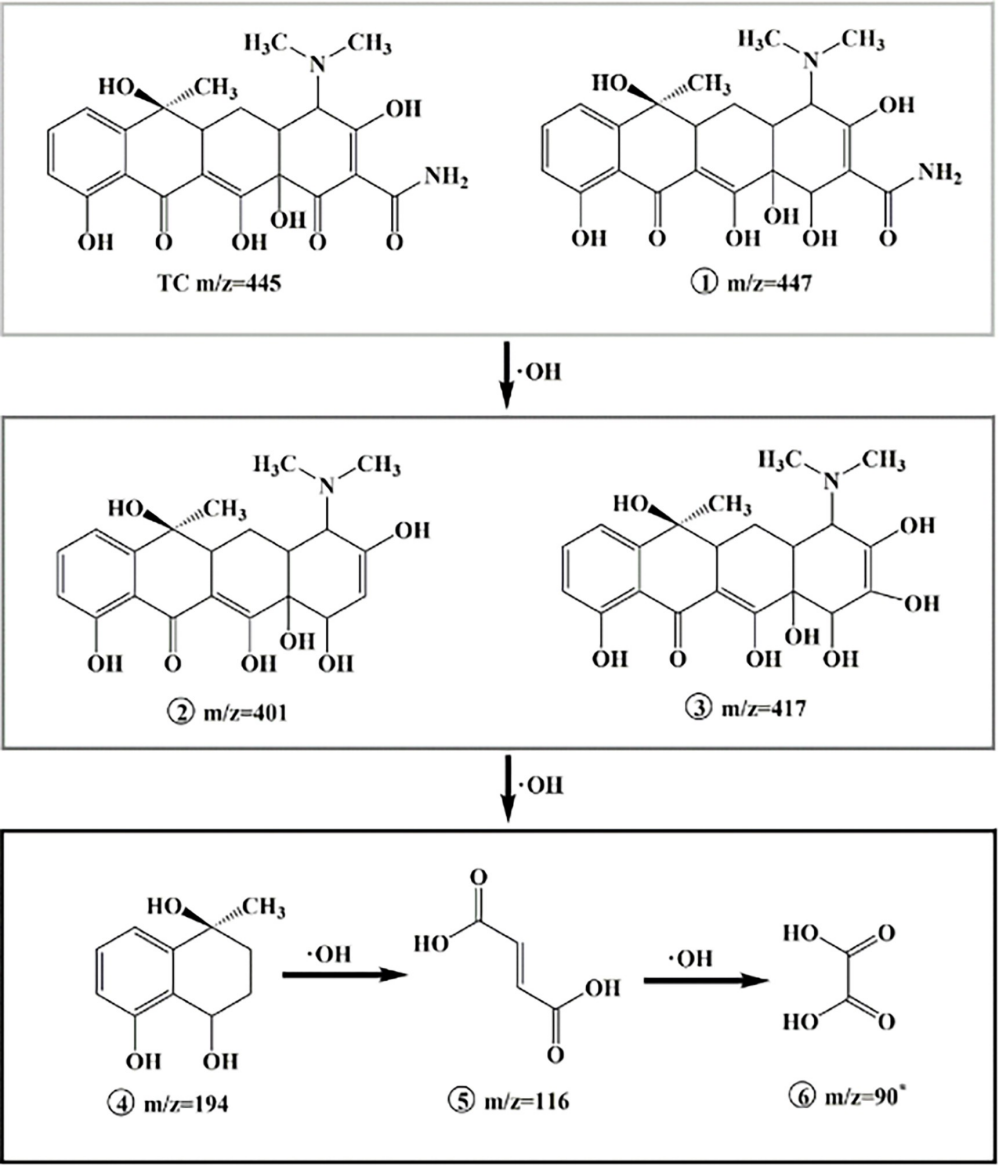
Proposed degradation pathway of TC by Graphite-UiO-66(Zr)/Ti electrode. (*: proposed intermediate).

In order to further understand the TC removal mechanism by Ui Graphite-UiO-66(Zr)/Ti electrode, the generated intermediates during TC degradation were detected by LC-MS and the main intermediates are summarized in S4 Fig. According to the detected intermediates and related research literatures [2, 5, 21, 47], the possible degradation pathway was proposed. As depicted in Fig 8, TC (m/z = 445) was converted to product ① (m/z = 447) through hydrogenation reaction, then they were further degraded to intermediates ② (m/z = 401) and ③ (m/z = 417) [57]. After that, through the radical attack, it was fragmented into small fractions ④ (m/z = 194), ⑤ but-2-enedioic acid (m/z = 116) and ⑥ oxalic acid (m/z = 90, the proposed intermediate) before fully mineralization. Notably, some different intermediates such as m/z = 367, m/z = 351 and m/z = 298 were reported by Wang et al. [5] who used the Ti/Ti_4_O_7_ electrode. However, these intermediates were not observed in our study, and the main reason could be the different attack mode of radicals produced by different electrodes or the instability of the intermediates produced in our solution.

### Stability of electrode

**Fig 9:**
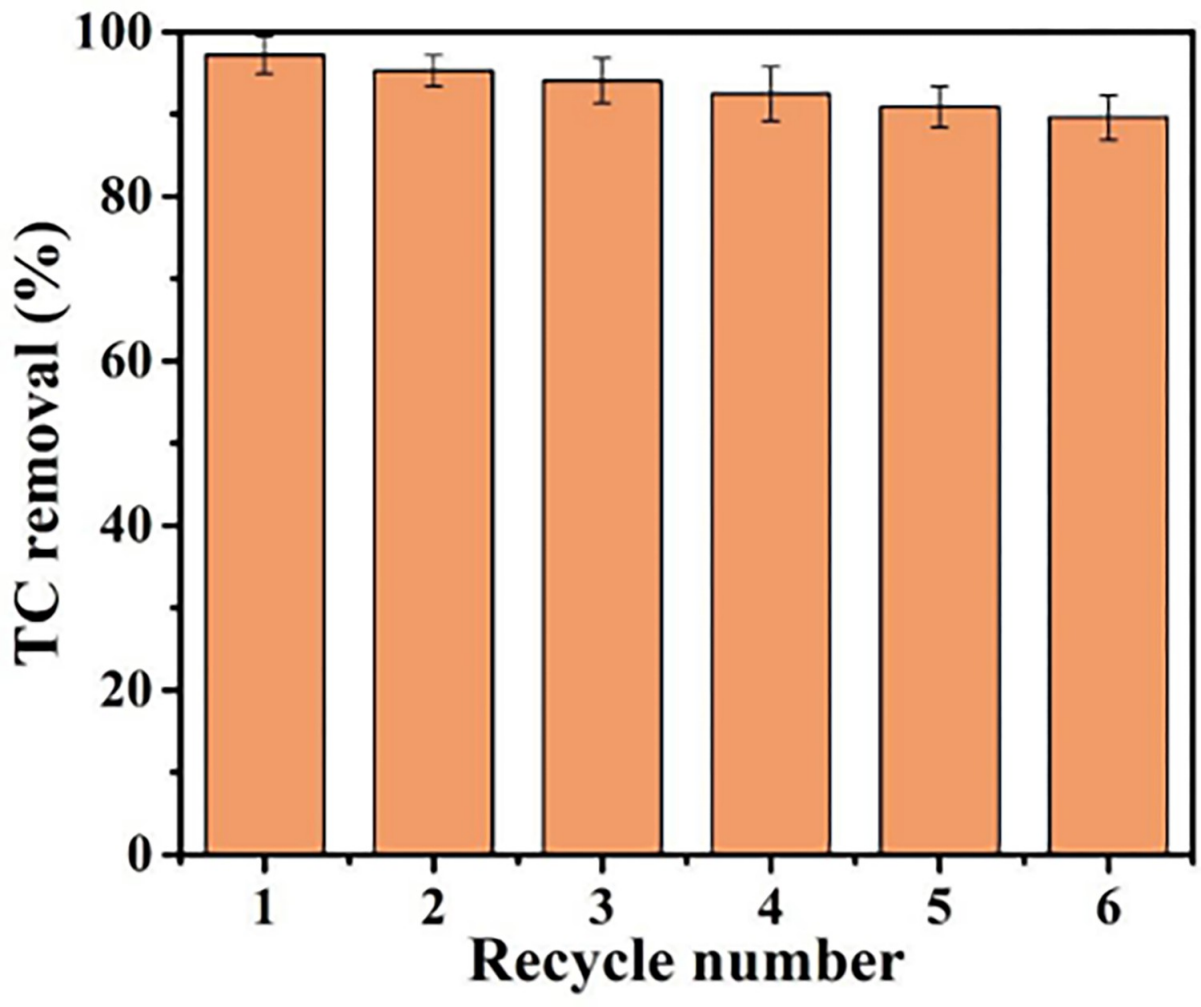
The removal rate of TC for 6 cycles. (100 mg/L, 0.1 M Na_2_SO_4_, 20 mA/cm^2^, 240 min).

Electrode stability was a crucial property for real application [18]. The consecutive six-cycle tests of Graphite-UiO-66(Zr)/Ti electrode for the TC removal were conducted. Fig 9 revealed that the TC removal remained at 89.6% ± 2.7% with the increasing cycles for Graphite-UiO-66(Zr)/Ti electrode under current density of 20 mA/cm^2^. To further determine the stability of the Graphite-UiO-66(Zr)/Ti electrode, we also characterized the Graphite-UiO-66(Zr)/Ti electrode before and after electrocatalysis. The FT-IR spectra (S5 Fig) and XRD patterns (S5 Fig) of Graphite-UiO-66(Zr)/Ti electrode negligibly changed after electrocatalysis, which revealed that the Graphite-UiO-66(Zr)/Ti electrode had an efficient electrochemical stability.

## Conclusions

In this work, a novel Graphite-UiO-66(Zr)/Ti electrode was successfully prepared and evaluated for electrochemical oxidation degradation of TC. The electrochemical performance tests indicated the Graphite-UiO-66(Zr)/Ti electrode had higher electrochemical oxidation activity, which achieved higher TC removal (98.1% ± 1.5%) than Ti plate (65.2% ± 3.5%), Graphite-MIL-53(Al)/Ti electrode (79.5% ± 2.9%) and Graphite-MIL-100(Fe)/Ti electrode (89.0% ± 2.6%). The influence of operating condition was also systematically studied, and the optimized condition was pH 5.0, 20 mA/cm^2^ current density and 0.1 M electrolyte (Na_2_SO_4_). Through the liquid chromatography mass spectrometry (LC-MS), the TC degradation pathway by Graphite-UiO-66(Zr)/Ti electrode oxidation was proposed. Under the •OH free radical oxidative decomposition effect, the double bond, phenolic group and amine group of TC were attacked. TC was transformed into intermediate product ① (m/z = 447), then was further degraded to intermediates ② (m/z = 401) and ③ (m/z = 417). The latter was fragmented into small fractions ④ (m/z = 194), ⑤ but-2-enedioic acid (m/z = 116) and ⑥ oxalic acid (m/z = 90, the proposed intermediate). In addition, TC removal remained at 89.6% ± 2.7% in the sixth cycle of operation, which confirmed the efficient reusability and stability for antibiotics removal from water.

## Supporting information

S1 Fig Photo of anodes. (a) Graphite-MIL-53(Al)/Ti. (b) Graphite-MIL-100(Fe)/Ti. (c) Graphite-UiO-66(Zr)/Ti.
(TIF)

S2 Fig XPS survey spectra of anodes. (a) Graphite-MIL-53(Al)/Ti. (b) Graphite-MIL-100(Fe)/Ti. (c) Graphite-UiO-66(Zr)/Ti.
(TIF)

S3 Fig Effect on adsorption capacity (Q_t_) by Graphite-UiO-66(Zr)/Ti under different pHs without electricity. (TIF)

S4 Fig LC-MS spectra of the transformation products of TC by Graphite-UiO-66(Zr)/Ti electrode. (TIF)

S5 Fig Characterization of Graphite-UiO-66(Zr)/Ti electrode before and after electrocatalysis. (a) FTIR spectra. (b) XRD pattern.
(TIF)
