# Supplementary figures and images for: Preparation of Graphite-UiO-66(Zr)/Ti electrode for efficient electrochemical oxidation of tetracycline in water
Source: PLoS One. 2022 Aug 9;17(8):e0271075. doi: 10.1371/journal.pone.0271075 (PMC9362921; doi:10.1371/journal.pone.0271075)

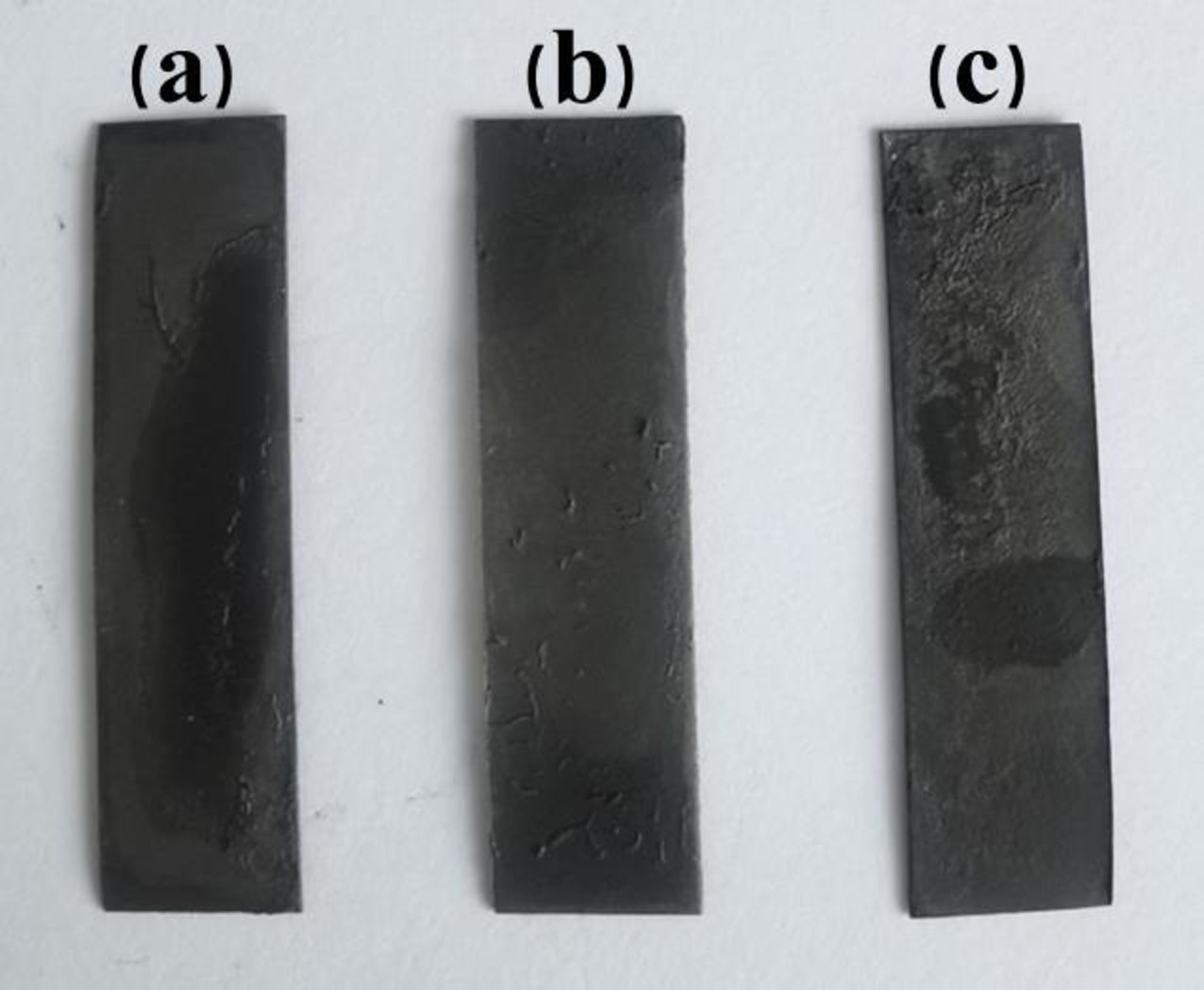

Supplement: S1 Fig — (a) Graphite-MIL-53(Al)/Ti. (b) Graphite-MIL-100(Fe)/Ti. (c) Graphite-UiO-66(Zr)/Ti. (TIF) [file pone.0271075.s001.tif]

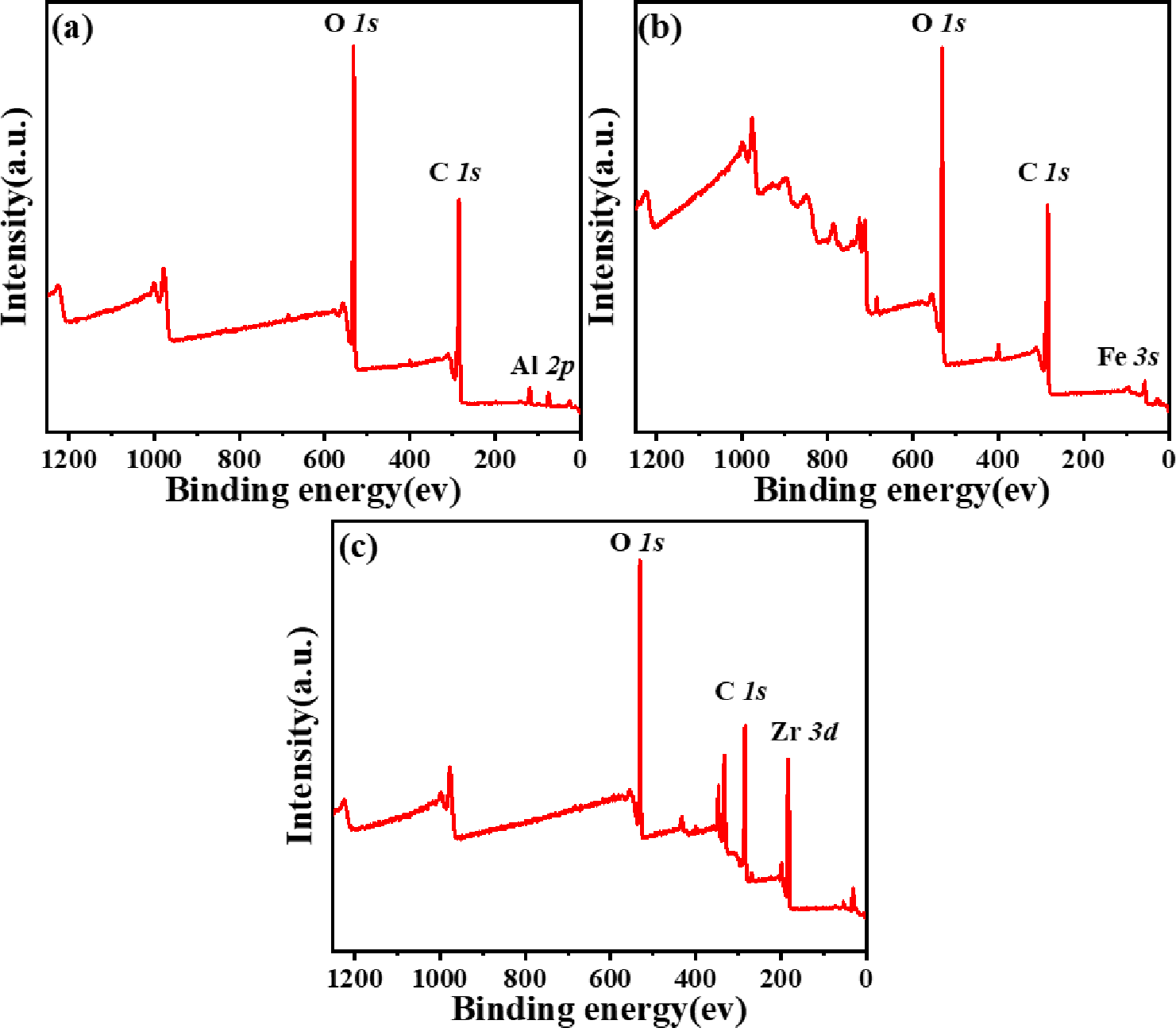

Supplement: S2 Fig — (a) Graphite-MIL-53(Al)/Ti. (b) Graphite-MIL-100(Fe)/Ti. (c) Graphite-UiO-66(Zr)/Ti. (TIF) [file pone.0271075.s002.tif]

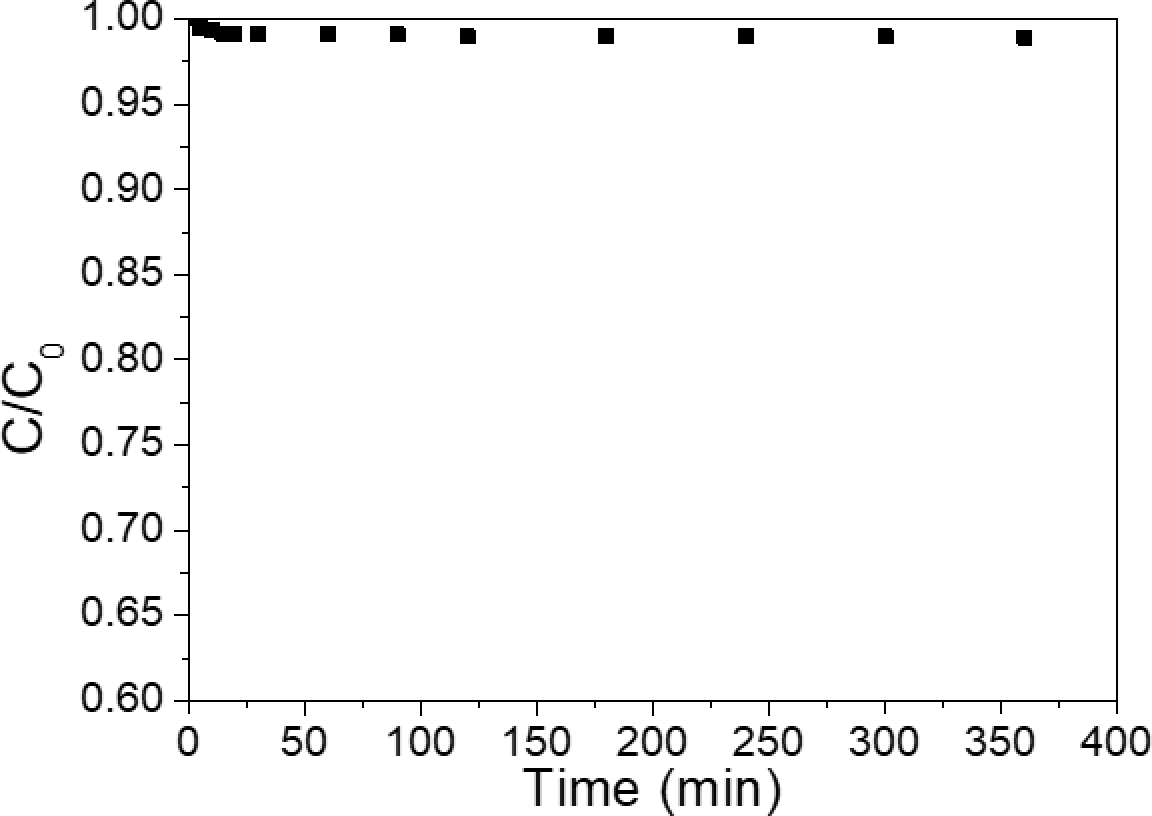

Supplement: S3 Fig — (TIF) [file pone.0271075.s003.tif]

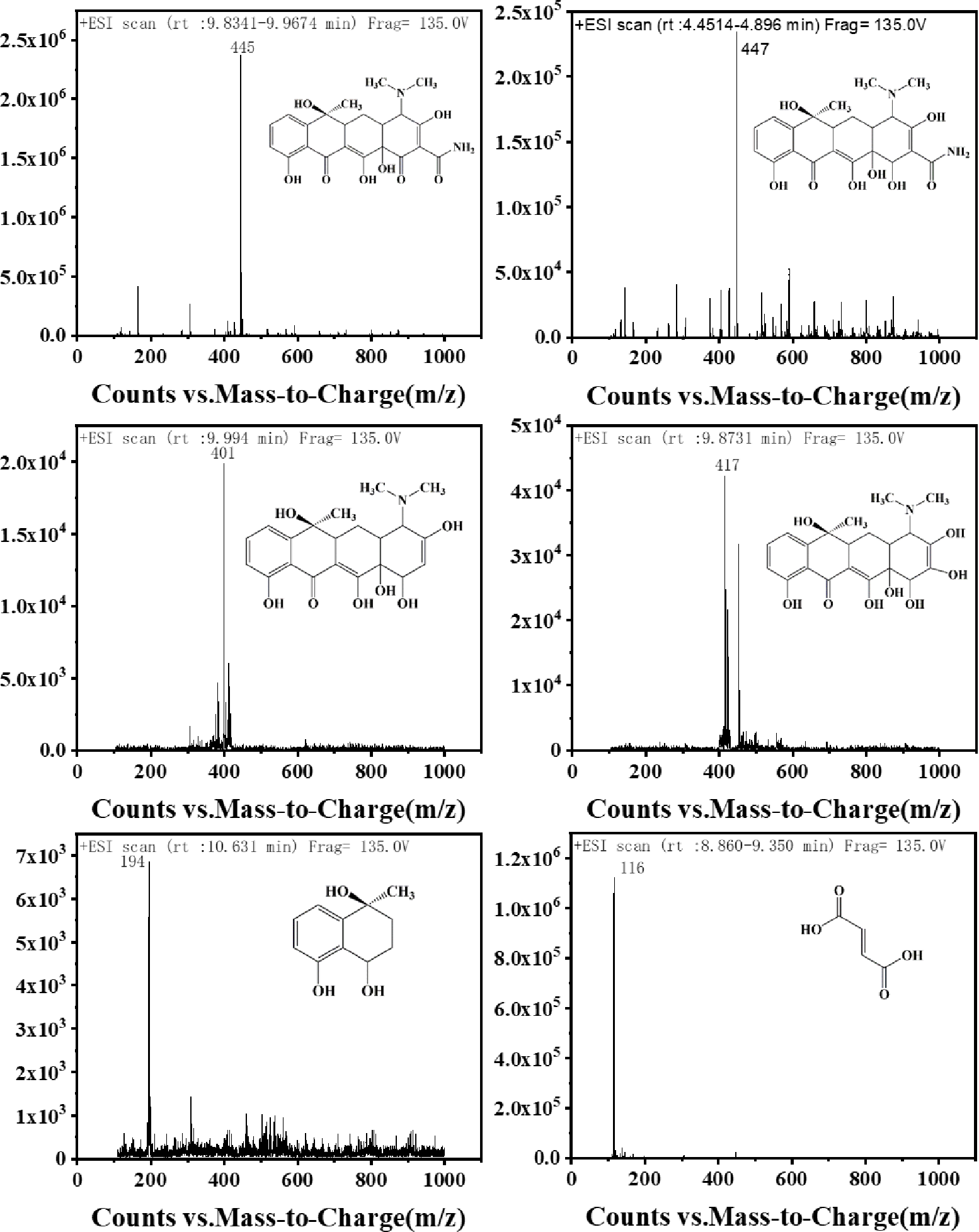

Supplement: S4 Fig — (TIF) [file pone.0271075.s004.tif]

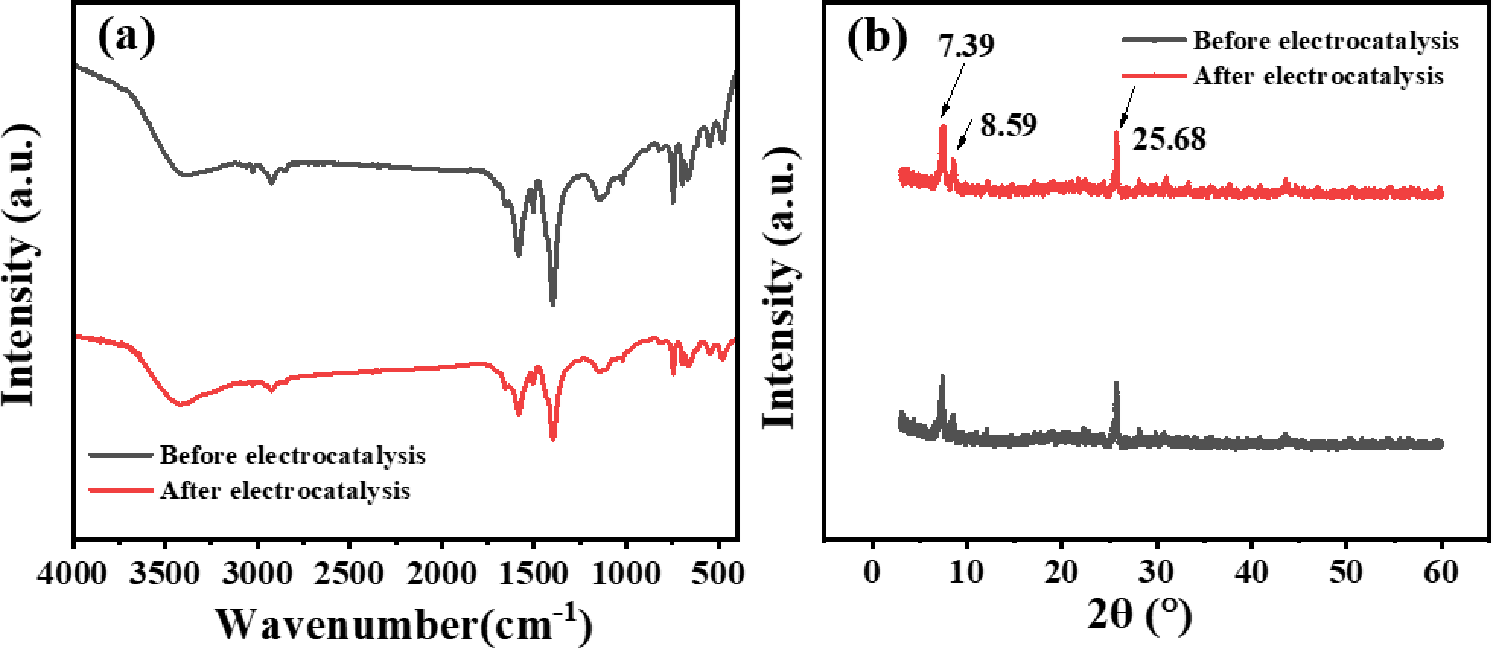

Supplement: S5 Fig — (a) FTIR spectra. (b) XRD pattern. (TIF) [file pone.0271075.s005.tif]
